# Supplementary material for: Structural basis for selectivity and antagonism in extracellular GPCR-nanobodies
Source: Nat Commun. 2024 May 30;15:4611. doi: 10.1038/s41467-024-49000-x (PMC11139983; doi:10.1038/s41467-024-49000-x)
Supplement: Supplementary file 1 — Supplementary Information [file 41467_2024_49000_MOESM1_ESM.pdf]

Supplementary Information for "Structural Basis for Selectivity and Antagonism in Extracellular GPCR-Nanobodies"

Supplementary Information:

Contruct	log CXCL11 EC_{50} (M)	log VUN701 IC_{50} (M)	[CXCL11] in Comp. (nM)	log VUN701 K_B (M)
WT ACKR3	-8.54 ± 0.06	-6.98 ± 0.06	10	-7.63 ± 0.37
WT CXCR3	-7.63 ± 0.05	N/A	140	N/A

Supplementary Table 1:
Summary of EC_{50}, IC_{50}, and K_B values for ACKR3 and CXCR3 in BRET-based β-arrestin2 recruitment assays with CXCL11.

Contruct	log CXCL12 EC_{50} (M)	log VUN701 IC_{50} (M)	[CXCL12] in Comp. (nM)	log VUN701 K_B (M)
WT ACKR3	-9.66 ± 0.07	-6.80 ± 0.08	3.3	-8.01 ± 0.11
WT CXCR4	-8.82 ± 0.18	N/A	10	N/A
ACKR3 Mutant				
D30A	N/D	N/A	N/A	N/A
K40A	-9.72 ± 0.15	-6.68 ± 0.08	3.3	-7.94 ± 0.15
D179A^{4x61}	-7.55 ± 0.15	N/D	N/A	N/A
E193A	-9.72 ± 0.14	-6.91 ± 0.07	3.3	-8.18 ± 0.15
R197A^{45x51}	N/D	N/A	N/A	N/A
F199A	-9.60 ± 0.15	-6.86 ± 0.10	3.3	-8.00 ± 0.17
E202A	N/D	N/A	N/A	N/A
K206A^{5x33}	-9.33 ± 0.20	-7.02 ± 0.08	3.3	-7.93 ± 0.19
E213A^{5x40}	-9.19 ± 0.16	-7.18 ± 0.18	3.3	-7.97 ± 0.24
D275A^{6x58}	-8.54 ± 0.19	-5.18 ± 0.15	33	-6.28 ± 0.23
F285A	-9.19 ± 0.16	-4.78 ± 0.12	3.3	-5.57 ± 0.17
R288A^{7x25}	-9.61 ± 0.21	-6.73 ± 0.07	3.3	-7.89 ± 0.19
E290A^{7x27}	-9.34 ± 0.17	-5.30 ± 0.20	3.3	-6.22 ± 0.27
F294A^{7x31}	-9.67 ± 0.16	-6.77 ± 0.08	3.3	-7.99 ± 0.16
A291C^{6x54}	-9.25 ± 0.11	N/D	N/A	N/D
L293C^{7x30}	-9.02 ± 0.07	N/D	N/A	N/D
A271C^{6x54} L293C^{7x30}	-9.19 ± 0.24	-5.79 ± 0.15	3.3	-6.72 ± 0.30

Supplementary Table 2:
Summary of EC_{50}, IC_{50}, and K_B values for ACKR3, CXCR4, and ACKR3 mutants in BRET-based β-arrestin2 recruitment assays with CXCL12.

log [VUN701]	log EC_{50} (M)	log VUN701 K_B (M)	95% CI
0	-9.30 ± 0.07	-7.740	-7.802 to -7.681
-7.41	-8.85 ± 0.06		
-6.81	-8.16 ± 0.06		
-6.20	-7.66 ± 0.06		
-5.60	-7.13 ± 0.06		
-5.00	-6.45 ± 0.07		

Supplementary Table 3:
Summary of EC_{50} values and the K_B value calculated in ACKR3-VUN701 Schild analysis.

**Experimental constraints**		
Distance constraints for each monomer		
Residues		
Long		625
Medium [1<(i–j) ≤ 5]		109
Sequential [(i–j) = 1]		354
Intraresidue [i=j]		373
Total		1462
Dihedral angle constraints (ϕ and ψ)		199
Number of restrains per residue		13.7
Number of long-range restrains per residue		5.2
**Average atomic R.M.S.D. to the mean structure (Å)**		
Residues		1-51, 56-100 & 106-120
Backbone (C^{α}, C′, N)		0.62 ± 0.08
Heavy atoms		1.09 ± 0.08
**Deviations from idealized covalent geometry**		
Bond lengths	RMSD (Å)	0.016
Torsion angle violations	RMSD (°)	1.2
**Constraint violations**		
NOE distance	Number > 0.5 Å[a]	0 ± 0
NOE distance	RMSD (Å)	0.018 ± 0.001
Torsion-angle violations	Number > 5 °[b]	0.0 ± 0
Torsion-angle violations	RMSD (°)	0.746 ± 0.080
**Global quality scores (raw/Z score)**[c]		
Verify3D		0.32 / -2.25
Prosall		0.46 / -0.79
PROCHECK (ϕ–ψ)[d]		-0.41 / -1.30
PROCHECK (all)[d]		-0.34 / -2.01
MolProbity clash score		11.01 / -0.36
**Ramachandran statistics (% of all residues)**[e]		
Most favored		94.4
Additionally allowed		5.4
Generously allowed		0.2
Disallowed		0

Supplementary Table 4:
Summary of statistics of 20 VUN701 conformers.

Contruct	T_M (˚C)
VUN701 Mutant	
WT	71.0
T28A	70.7
F29A	71.1
S30A	71.1
L31A	66.4
H32A	70.3
D56A	69.7
K100A	69.3
I101A	66.8
G102A	69.0
R103A	71.6
D104A	68.5
T105A	70.1
F106A	65.8
R107A	70.1
ΔCDR1	59.0
ΔCDR3	70.9

Supplementary Table 5:
Melting temperatures (T_M) for VUN701 and VUN701 mutants measured by nanoDSF; n=3.

Contruct	log VUN701 IC_{50} (M)	[CXCL12] in Comp. (nM)	log VUN701 K_B (M)
VUN701 Mutant			
WT	-6.60 ± 0.05	3.3	-7.77 ± 0.11
T28A	-6.97 ± 0.06	3.3	-8.14 ± 0.12
F29A	-6.92 ± 0.08	3.3	-8.09 ± 0.14
S30A	-6.74 ± 0.06	3.3	-7.91 ± 0.11
L31A	-6.46 ± 0.06	3.3	-7.63 ± 0.12
H32A	-5.53 ± 0.07	3.3	-6.70 ± 0.13
D56A	-5.19 ± 0.07	3.3	-6.36 ± 0.13
K100A	< -4	3.3	N/A
I101A	-6.75 ± 0.09	3.3	-7.92 ± 0.15
G102A	-7.16 ± 0.09	3.3	-8.33 ± 0.14
R103A	-6.21 ± 0.10	3.3	-7.38 ± 0.16
D104A	-7.21 ± 0.10	3.3	-8.38 ± 0.15
T105A	-6.90 ± 0.07	3.3	-8.08 ± 0.12
F106A	-5.85 ± 0.10	3.3	-7.02 ± 0.16
R107A	-6.95 ± 0.09	3.3	-8.12 ± 0.15

Supplementary Table 6:
Summary of IC_{50} and K_B values for VUN701 and VUN701 mutants in BRET based β-arrestin2 recruitment assays with ACKR3.

Contruct	log CXCL12 EC_{50} (M)	log VUN701 IC_{50} (M)	[CXCL11] in Comp. (nM)	log VUN701 K_B (M)
WT CXCR3	-7.63 ± 0.06	N/A	140	N/A
<u>CXCR3 Mutant</u>				
$R288F^{ECL3}$	-7.69 ± 0.06	N/A	140	N/A
ECL3 swap	-7.81 ± 0.07	N/A	140	N/A

Supplementary Table 7:
Summary of EC_{50}, IC_{50}, and K_B values for CXCR3 and CXCR3 mutants in BRET-based β-arrestin2 recruitment assays with CXCL11.

Contruct	log CXCL12 EC_{50} (M)	log VUN701 IC_{50} (M)	[CXCL12] in Comp. (nM)	log VUN701 K_B (M)
WT CXCR4	-8.43 ± 0.07	N/A	10	N/A
<u>CXCR4 Mutant</u>				
Q272F^{ECL3}	-8.51 ± 0.10	N/A	10	N/A
ECL3 swap	-8.87 ± 0.19	N/A	10	N/A

Supplementary Table 8:
Summary of EC_{50}, IC_{50}, and K_B values for CXCR4 and CXCR4 mutants in BRET-based β-arrestin2 recruitment assays with CXCL12.

Contruct	log Chemokine* EC_{50} (M)	log ΔCDR1 IC_{50} (M)	[Chemokine*] in Comp. (nM)	log ΔCDR1 K_B (M)
ACKR3	-9.62 ± 0.06	-6.35 ± 0.08	3.3	-7.52 ± 0.01
CXCR3	-7.73 ± 0.09	N/A	140	N/A
CXCR4	-8.50 ± 0.09	-5.40 ± 0.11	10	-6.02 ± 0.02

Supplementary Table 9:
Summary of EC_{50}, IC_{50}, and K_B values for ACKR3, CXCR3, and CXCR4 in BRET-based β-arrestin2 competition assays with and VUN701 ΔCDR1. *The chemokine CXCL11 was used to assay CXCR3, while CXCL12 was used to assay ACKR3 and CXCR4.

	General Information			Lipid Composition						Ions	
Simulation	Total Atoms	Waters (TIP3)	Salt Conc.	CHL1	DOPC	DDPC	POPE	POPA	POPS	Sodium	Chloride
ACKR3-VUN701	119708	29705	150 mM	48	64	16	48	16	8	103	81
ACKR3-CXCL12	111821	27297	150 mM	48	64	16	48	16	8	88	74

Supplementary Table 10:
Summary of molecular dynamics system setup.

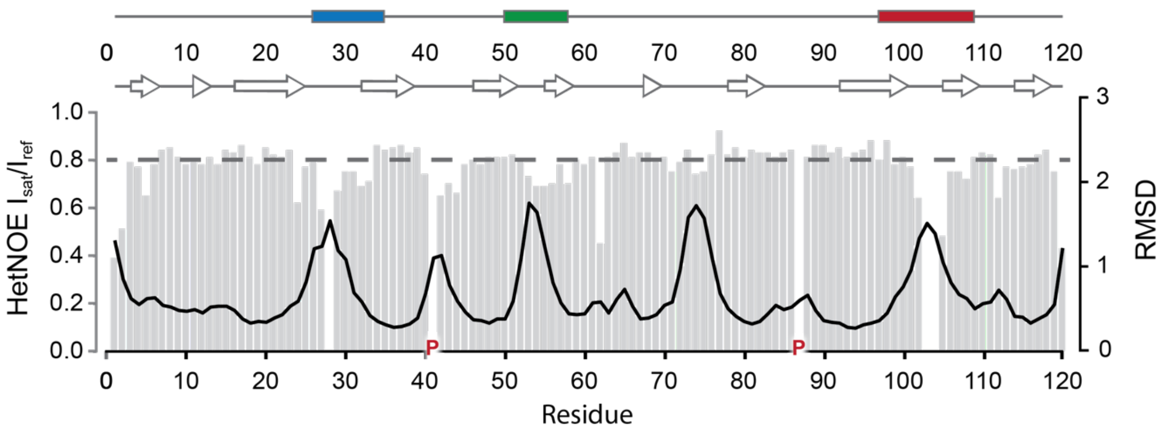

Supplementary Figure 1:
Plot of {^{1}H}-^{15}N heteronuclear NOE experiment of 1 mM [^{1}H-^{15}N]-VUN701 (grey bars) and structural ensemble RMSD (black line). The sequence and secondary structure of VUN701 are displayed above the plot. Proline residues are indicated with a red P. Source data are provided as a Source Data file.

	CDR1	CDR2	CDR3
**VUN701**	GSTFSLHLMG	GSGGD	AQQKIGRDTFRDY
**ΔCDR1**	GS**GSGS**HLMG	GSGGD	AQQKIGRDTFRDY
**ΔCDR2**	GSTFSLHLMG	GSGG**A**	AQQKIGRDTFRDY
**ΔCDR3**	GSTFSLHLMG	GSGGD	AQ**---GSGG--**DY

Supplementary Figure 2:
Sequence alignment of the three VUN701 CDRs with the CDRs of each ΔCDR constructs. Mutated residues are colored in black.

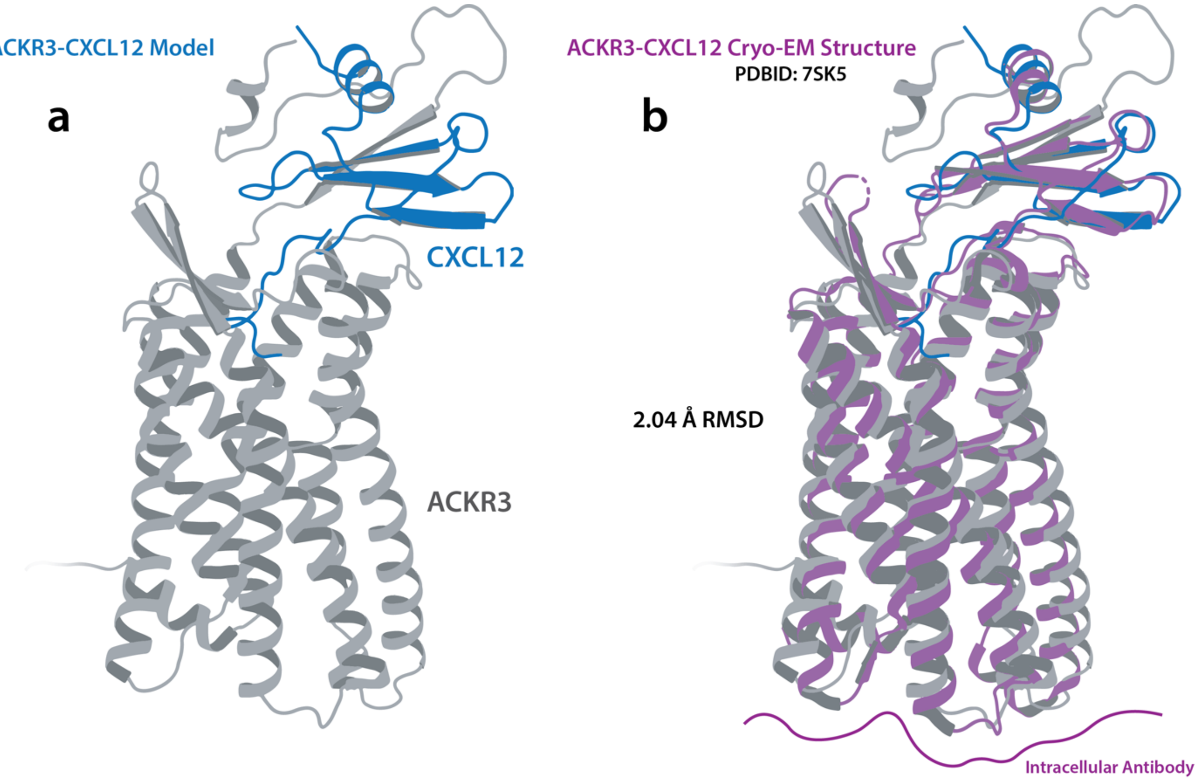

Supplementary Figure 3:
An ACKR3-CXCL12 model was generated from three rounds of AlphaFold 2 design without templates. Top models from each were placed into synthetic membrane with implicit solvent and underwent 1 µsec simulations in Gromacs 2021.2. (A) Highest scoring ACKR3-CXCL12 model is shown at 500 ns. (B) Overlay of the generated ACKR3-CXCL12 model with the recently solved ACKR3-CXCL12-Fab-Nb Cryo-EM structure from Yen, Y. et al. (7SK5) [20]. A 2.04 Angstrom backbone RMSD was calculated between the two structures. Differences in structure primarily occur at the intracellular pocket due to the addition of a stabilizing antibody in the Cryo-EM structure which is not present in the molecular modeling.

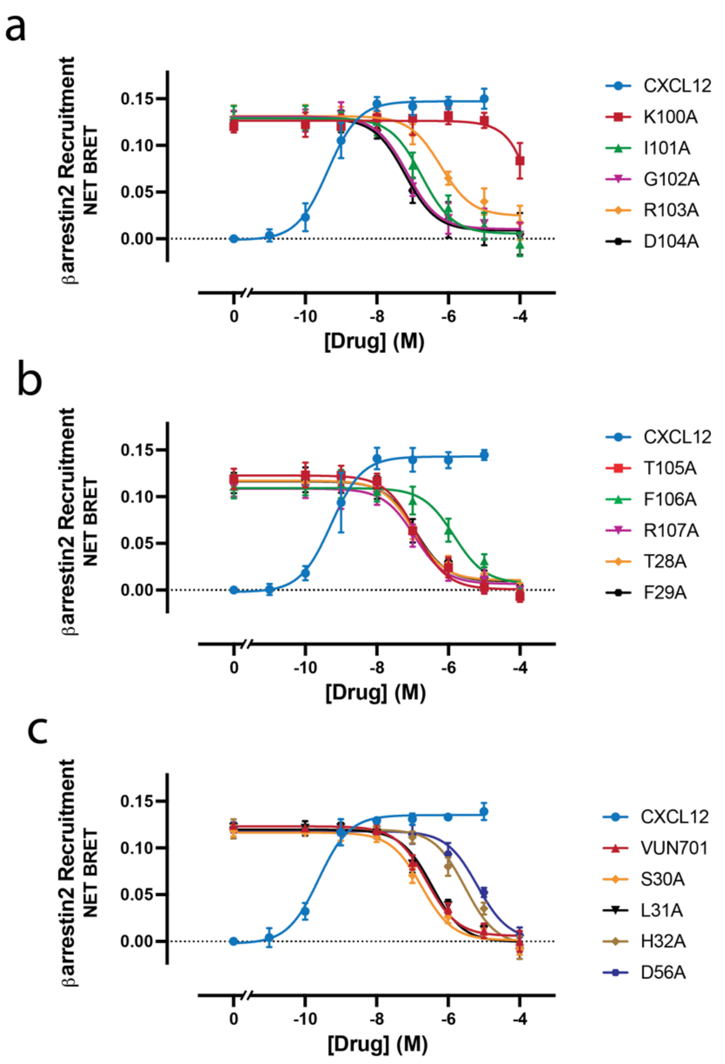

Supplementary Figure 4:
ACKR3 BRET-based β-arrestin2 competition assay of 3.3 nM CXCL12 with increasing VUN701 variants K100A, I101A, G102A, R103A, D104A (A); T105A, F106A, R107A, T28A, F29A (B); S30A, L31A, H32A, D56A (C). N = 3 biologically independent experiments plotted as mean +/- SEM. Source data are provided as a Source Data file.

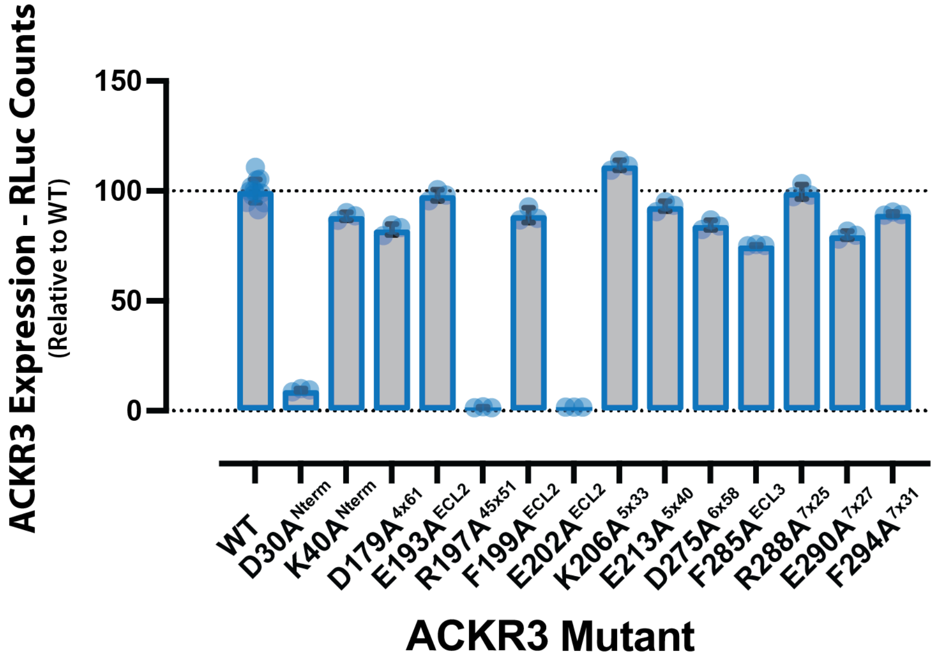

Supplementary Figure 5:
Expression levels of each ACKR3 mutant used in this study. Expression levels were quantified by Rluc counts measured in each BRET assay and normalized to the level of WT expression. N = 3 biologically independent experiments plotted as mean +/- SEM. Source data are provided as a Source Data file.

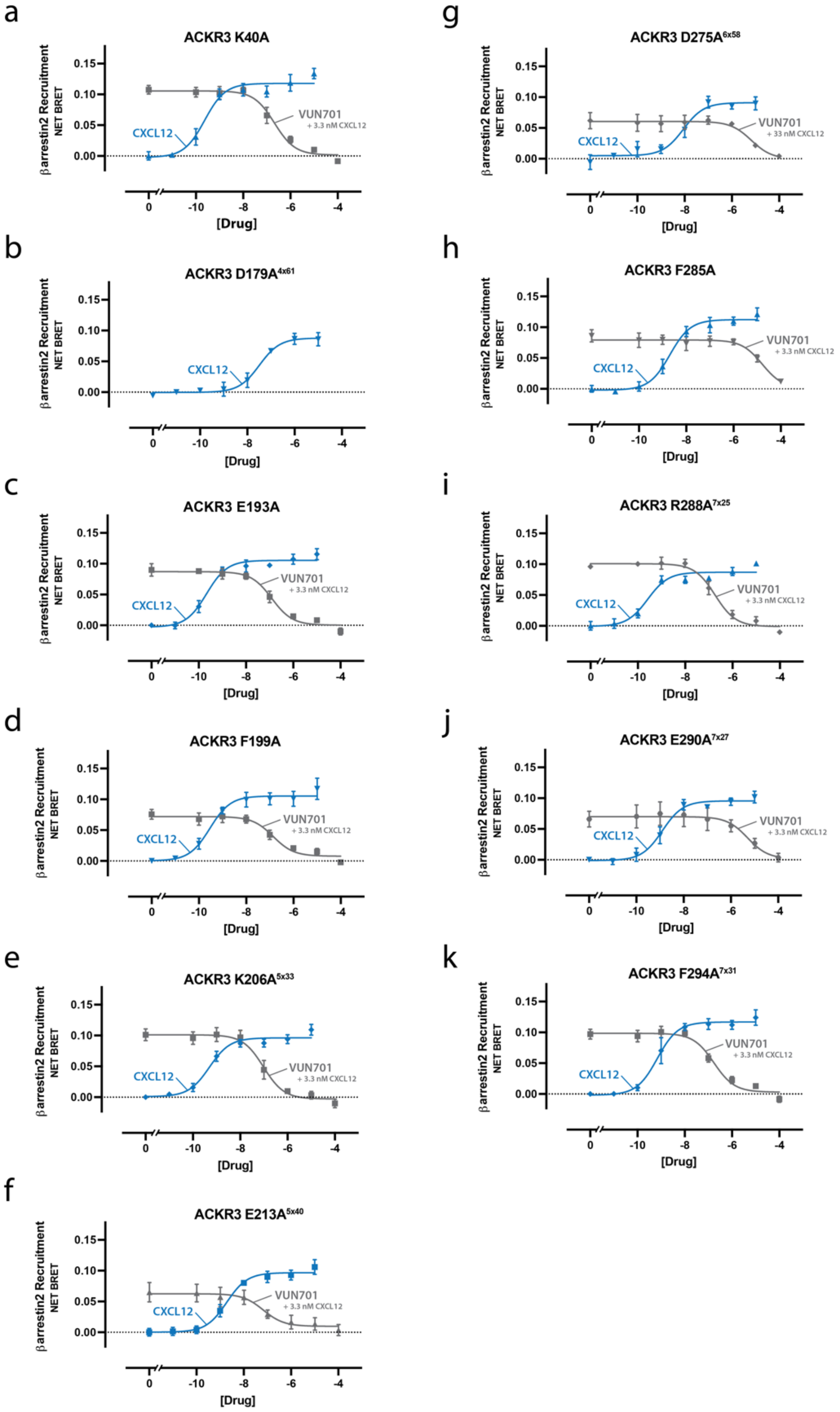

Supplementary Figure 6:

BRET-based β-arrestin2 recruitment assay of ACKR3 mutant K40A (A), D179A (B), E193A (C), F199A (D), K206A (E), E213A (F), D275A (G), F285A (H), R288A (I), E290A (J), or F294A (K) with endogenous ligand CXCL12 (blue) or with increasing VUN701 in the presence of fixed

CXCL12 (grey). N = 3 biologically independent experiments plotted as mean +/- SEM. Source data are provided as a Source Data file.

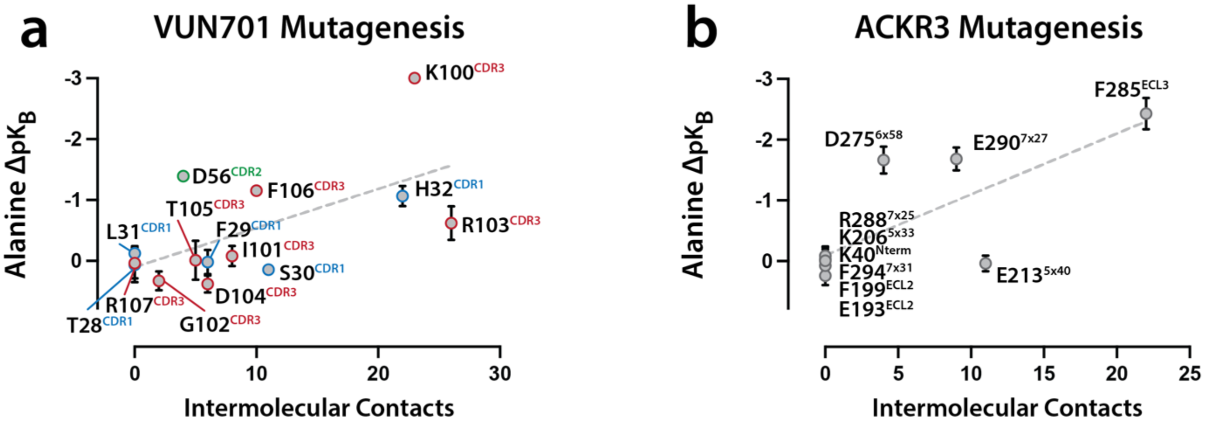

Supplementary Figure 7:

Plot of the number of intermolecular contacts in the ACKR3-VUN701 model made at each residue probed in the β-arrestin2 recruitment assay against that residues' corresponding shift in K_B for VUN701 (A) and ACKR3 (B). Intermolecular contacts determined using the Protein Contact Atlas. VUN701 plot colored according to CDR as in Figure 2a. Source data are provided as a Source Data file.

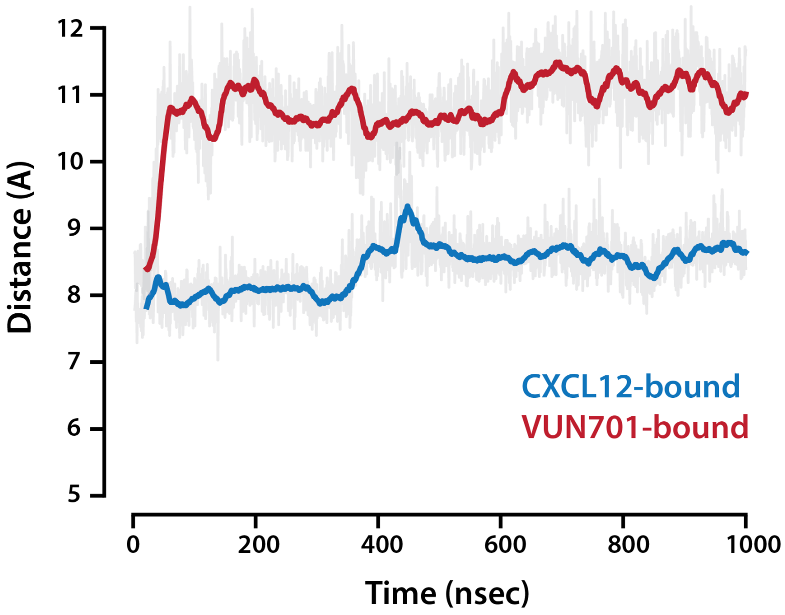

Supplementary Figure 8:
Plot of the distance (in Angstroms) between the Cα carbons of ACKR3's D90^{2x50} and N311^{7x49} throughout the MD simulation time course (nanoseconds).

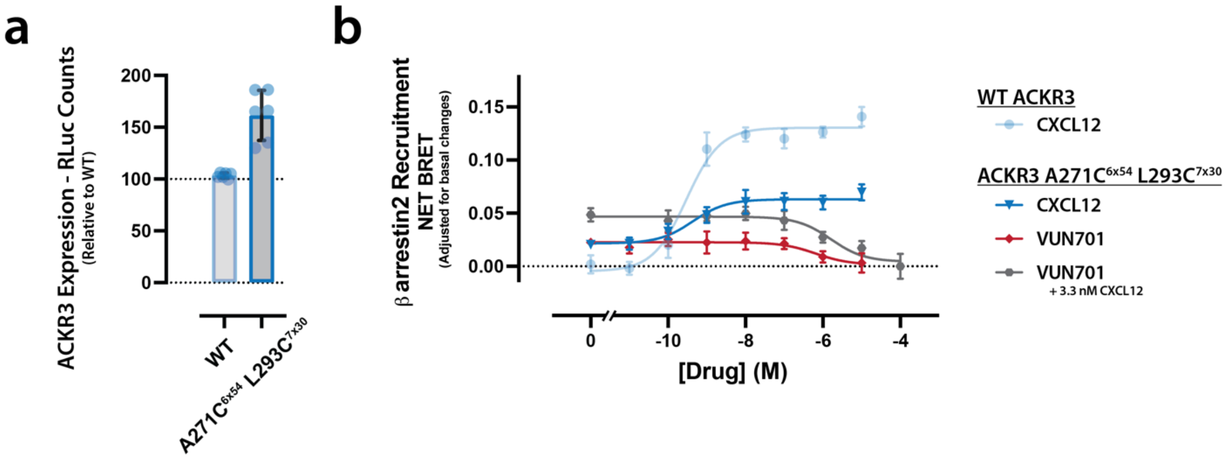

Supplementary Figure 9:
A) Expression levels of ACKR3 A271C^{6x54} L293C^{7x30} compared to WT ACKR3. Expression levels were quantified by Rluc counts measured in each BRET assay and normalized to the level of WT expression. (B) BRET-based β-arrestin2 recruitment assay as in Figure 4d with increasing CXCL12 (blue), VUN701 (red), or VUN701 in the presence of 3.3 nM CXCL12 (grey). Adjusted for the increased basal β-arrestin2 recruitment of ACKR3 A271C^{6x54} L293C^{7x30} vs WT ACKR3. N = 3 biologically independent experiments plotted as mean +/- SEM. Source data are provided as a Source Data file.

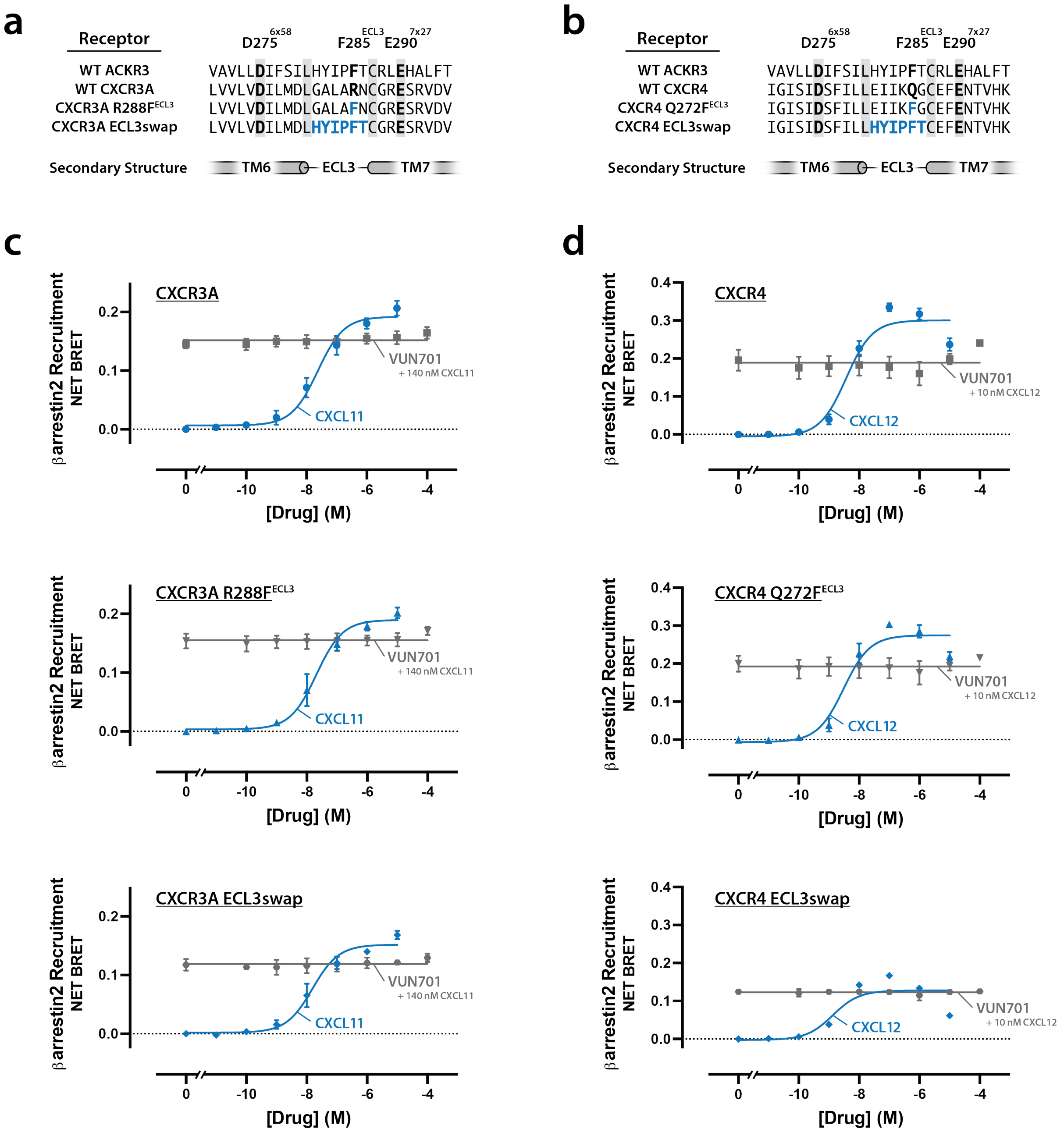

Supplementary Figure 10:
(A) Sequence overlay comparing the ECL3 region of ACKR3, CXCR3A, CXCR3A R288F, and CXCR3A ECL3swap mutant. (B) Sequence overlay comparing the ECL3 region of ACKR3, CXCR4, CXCR4 Q272F, and CXCR4 ECL3swap mutant. BRET-based β-arrestin2 recruitment assay with indicated chemokine (blue) and with VUN701 in the presence of indicated chemokine (grey). (C) WT CXCR3A (top), CXCR3A R288F^{ECL3} (middle), and a chimera of CXCR3A with ACKR3's ECL3 (bottom) with CXCL11. Competition experiments used 140 nM CXCL11. (D) WT CXCR4 (top), CXCR4 Q272F^{ECL3} (middle), and a chimera of CXCR4 with ACKR3's ECL3 (bottom) with CXCL12. Competition experiments used 10 nM CXCL12. N = 3 biologically independent experiments plotted as mean +/- SEM. Source data are provided as a Source Data file.

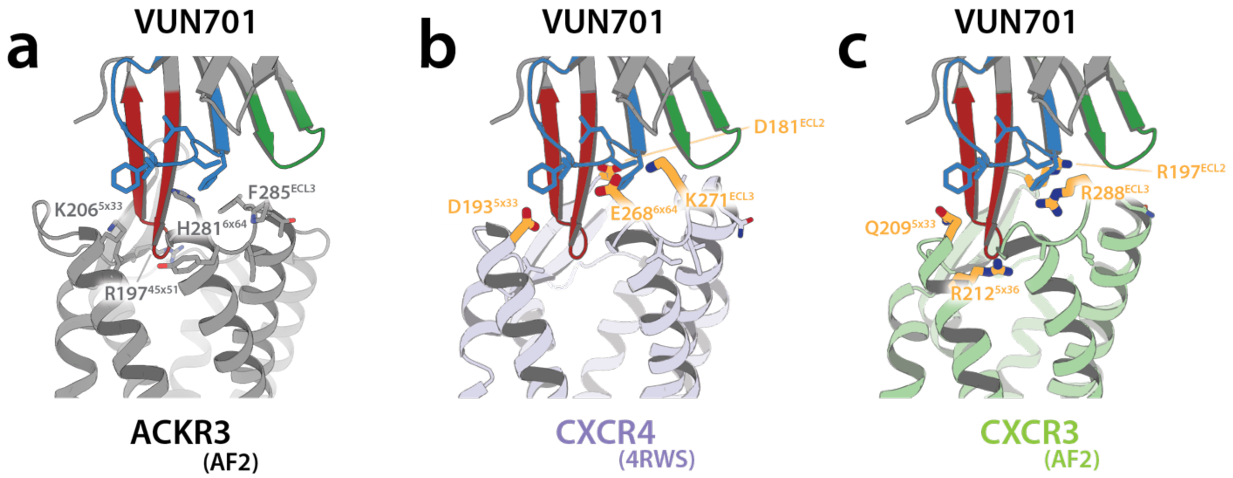

Supplementary Figure 11:

A) ACKR3-VUN701 model outlining interactions between VUN701's CDR1 and ACKR3's TM5, ECL2, and ECL3. Superimposition of VUN701 with the B) CXCR4 structure (PDBID: 4RWS – purple) and C) CXCR3 AF model (green) showing steric clashes between charged residues (yellow) with VUN701.
