# Supplementary material for: Structural basis for selectivity and antagonism in extracellular GPCR-nanobodies
Source: Nat Commun. 2024 May 30;15:4611. doi: 10.1038/s41467-024-49000-x (PMC11139983; doi:10.1038/s41467-024-49000-x)
Supplement: Supplementary file 3 — Description of Additional Supplementary Files [file 41467_2024_49000_MOESM3_ESM.pdf]

Description of Additional Supplementary Files

Supplementary Data 1:
Classification of CDR3 conformation in all solved nanobody structures.

Supplementary Data 2:
Classification of CDR3 conformation in GPCR-targeting nanobodies. Nanobody name, target, and epitope provided.

Supplementary Data 3:
Primer sequences used in the manuscript and the associated PCR method/kit used for mutagenesis/cloning.

Supplementary Data 4:
Compressed folder containing the representative AlphaFold 2 models of ACKR3 with VUN701 or CXCL12 before MD simulations.

Supplementary Data 5:
Compressed folder containing the representative AlphaFold 2 models of ACKR3 with VUN701 or CXCL12 after MD simulations (500 nanoseconds).

Supplementary Data 6:
The reproducibility checklist for molecular dynamics simulations conducted in this manuscript.
