# Supplementary material for: Structural basis for selectivity and antagonism in extracellular GPCR-nanobodies
Source: Nat Commun. 2024 May 30;15:4611. doi: 10.1038/s41467-024-49000-x (PMC11139983; doi:10.1038/s41467-024-49000-x)
Supplement: Supplementary file 9 — Supplementary Data 6 [file 41467_2024_49000_MOESM9_ESM.docx]

| **Reliability and reproducibility checklist for molecular dynamics simulations**  ***All boxes must be marked YES by acceptance unless an N/A option is available** | | | **Yes** | **N/A** | **Response  (Please state where this information can be found in the text)** |
| --- | --- | --- | --- | --- | --- |
| **1. Convergence of simulations and analysis** | | | | | |
| 1a. Is an evaluation presented in the text to show that the property being measured has equilibrated in the simulations (*e.g.* time-course analysis)? | | |  |  | This information has been added as Supplementary Figure 8. |
| 1b. Then, is it described in the text how simulations are split into equilibration and production runs and how much data were analyzed from production runs? | | |  |  | This information is provided in the “ACKR3 Molecular Modeling” section of the Methods. All MD simulations were minimized, equilibrated, and run according to reference 45. |
| 1c. Are there at least 3 simulations per simulation condition with statistical analysis? | | |  |  | ACKR3-VUN701 simulations were run in triplicate. Only one ACKR3-CXCL12 simulation was run, to confirm that simulations resembled the available CryoEM structure. All comparisons to the active ACKR3 in this manuscript are made to the CryoEM structure. |
| 1d. Is evidence provided in the text that the simulation results presented are independent of initial configuration? | | |  |  | This is shown in Supplementary Figure 8. This information is also given from the comparison of the input and output MD files (Supplementary Data 4 and 5). |
| **2. Connection to experiments** | | | | | |
| 2a. Are calculations provided that can connect to experiments (*e.g.* loss or gain in function from mutagenesis, binding assays, NMR chemical shifts, J-couplings, SAXS curves, interaction distances or FRET distances, structure factors, diffusion coefficients, bulk modulus and other mechanical properties, *etc*.)? | | |  |  | Alanine scanning in Figures 3c, 3d, and 3f show clear connection to MD results. This is specifically demonstrated in Supplementary Figure 7. |
| **3. Method choice** | | | | | |
| 3a. Is it described in the text what force field and water model are used and why? | | |  |  | This information is provided in the “ACKR3 Molecular Modeling” section of the Methods. All MD simulations were minimized, equilibrated, and run according to reference 45 and the standards of membrane proteins in Charmm-GUI. |
| 3b. Do simulations contain membranes, membrane proteins, intrinsically disordered proteins, glycans, nucleic acids, polymers, or cryptic ligand binding? | | |  |  | Simulations contain membranes and membrane proteins. |
|  | If 3b is **YES**, are enhanced sampling methods used? | |  |  | Response not needed if **N/A** |
|  | | If enhanced sampling methods are used, are the convergence criteria clearly stated? |  |  |  |
|  | If 3b is **YES**, is it explained in the text why or why not enhanced sampling methods are used? | |  |  |  |
| **4. Code and reproducibility** | | | | | |
| 4a. Is a table provided describing the system setup, such as simulation box dimensions, total number of atoms, total number of water molecules, salt concentration, lipid composition (number of molecules and type)? | | |  |  | Supplementary Table 10 is provided to describe system setup. |
| 4b. Is it described in the text what simulation and analysis software and which versions are used? | | |  |  | Simulation software and analysis is described in the “ACKR3 Molecular Modeling” section of the Methods. |
| 4c. Are initial coordinate and simulation input files and a coordinate file of the final output provided as supplementary files or in a public repository? | | |  |  | Input and output MD files are given as Supplementary Data 4 and 5. |
| 4d. Is there custom code or custom force field parameters? | | |  |  | Response not needed if **N/A** |
|  | If **YES**, are they provided as supplementary profiles or in a public repository? | |  |  |  |
