# Supplementary material for: Structural basis for selectivity and antagonism in extracellular GPCR-nanobodies
Source: Nat Commun. 2024 May 30;15:4611. doi: 10.1038/s41467-024-49000-x (PMC11139983; doi:10.1038/s41467-024-49000-x)
Supplement: Supplementary file 10 — Reporting Summary [file 41467_2024_49000_MOESM10_ESM.pdf]

nature portfolio

Corresponding author(s): Brian Volkman

Last updated by author(s): 10/22/2023

Reporting Summary

Nature Portfolio wishes to improve the reproducibility of the work that we publish. This form provides structure for consistency and transparency in reporting. For further information on Nature Portfolio policies, see our Editorial Policies and the Editorial Policy Checklist.

Statistics

For all statistical analyses, confirm that the following items are present in the figure legend, table legend, main text, or Methods section.

n/a	Confirmed	
☐	☒	The exact sample size (*n*) for each experimental group/condition, given as a discrete number and unit of measurement
☐	☒	A statement on whether measurements were taken from distinct samples or whether the same sample was measured repeatedly
☐	☒	The statistical test(s) used AND whether they are one- or two-sided<br>*Only common tests should be described solely by name; describe more complex techniques in the Methods section.*
☒	☐	A description of all covariates tested
☐	☒	A description of any assumptions or corrections, such as tests of normality and adjustment for multiple comparisons
☐	☒	A full description of the statistical parameters including central tendency (e.g. means) or other basic estimates (e.g. regression coefficient) AND variation (e.g. standard deviation) or associated estimates of uncertainty (e.g. confidence intervals)
☐	☒	For null hypothesis testing, the test statistic (e.g. *F*, *t*, *r*) with confidence intervals, effect sizes, degrees of freedom and *P* value noted<br>*Give P values as exact values whenever suitable.*
☒	☐	For Bayesian analysis, information on the choice of priors and Markov chain Monte Carlo settings
☒	☐	For hierarchical and complex designs, identification of the appropriate level for tests and full reporting of outcomes
☒	☐	Estimates of effect sizes (e.g. Cohen's *d*, Pearson's *r*), indicating how they were calculated

Our web collection on statistics for biologists contains articles on many of the points above.

Software and code

Policy information about availability of computer code

Data collection	NMR Data Collection: TopSpin 3.6;<br>NMR Data Analysis: NMRPipe 9.6, XEASY 1.3.13;<br>NMR Chemical Shift Assignment: GARANT 2.2;<br>NMR Data Refinement and Structure Calculation: CYANA 3.0;<br>NMR Geometry Optimization: XPLOR-NIH;<br>BRET Based Assays: MicroWIN2010 5.19;<br>Protein Modeling: ColabFold;<br>MD Setup and Production Runs: CHARMM-GUI, Gromacs 2021.2
Data analysis	Statistical analysis and curve fitting: GraphPad Prism 8.0.1;<br>Sequence alignment: GPCRdb;<br>Numerical data processing: Microsoft Excel 16.77;<br>Protein Contact Comparison: Protein Contact Atlas;<br>Protein Visualization: PyMOL 2.4.2;<br>CDR3 Comparison: Python3 Custom Code

For manuscripts utilizing custom algorithms or software that are central to the research but not yet described in published literature, software must be made available to editors and reviewers. We strongly encourage code deposition in a community repository (e.g. GitHub). See the Nature Portfolio guidelines for submitting code & software for further information.

Data

Policy information about availability of data

All manuscripts must include a data availability statement. This statement should provide the following information, where applicable:

- Accession codes, unique identifiers, or web links for publicly available datasets
- A description of any restrictions on data availability
- For clinical datasets or third party data, please ensure that the statement adheres to our policy

The NMR solution structure of VUN701 has been deposited in the PDB (rcsb.org) under the accession code 8UEK [https://doi.org/10.2210/pdb8UEK/pdb]. All other data supporting the findings of this study are available within the paper and its supplementary data files. PDB 7SK5 [https://doi.org/10.2210/pdb7SK5/pdb] was used for this study along with nanobody PDB files listed in Supplementary Data 1. The sequences of human ACKR3 and human CXCL12 were obtained from UniProt (P25106 and P48061). The GPCR database (gpcrdb.org) was used to generate sequence conservation plot in Figure5a/c. Source data are provided with this paper.

Research involving human participants, their data, or biological material

Policy information about studies with human participants or human data. See also policy information about sex, gender (identity/presentation), and sexual orientation and race, ethnicity and racism.

Reporting on sex and gender	N/A
Reporting on race, ethnicity, or other socially relevant groupings	N/A
Population characteristics	N/A
Recruitment	N/A
Ethics oversight	N/A

Note that full information on the approval of the study protocol must also be provided in the manuscript.

Field-specific reporting

Please select the one below that is the best fit for your research. If you are not sure, read the appropriate sections before making your selection.

☒ Life sciences ☐ Behavioural & social sciences ☐ Ecological, evolutionary & environmental sciences

For a reference copy of the document with all sections, see nature.com/documents/nr-reporting-summary-flat.pdf

Life sciences study design

All studies must disclose on these points even when the disclosure is negative.

Sample size	Assay replicates were chosen to demonstrate reproducibility and enable statistically significant results. No sample size calculations were performed. A sample size of three was used for all statistical analysis to conform with the standard in the field of molecular pharmacology.
Data exclusions	No data were excluded from analysis.
Replication	Positive controls were run with every BRET experiment. Occasional (1 of 10) plates contained replicate conditions to confirm data. All attempts at data replication were successful.
Randomization	Randomization was not relevant to this study. The results were not subject to investigator bias and no patients were used in this study.
Blinding	The investigators were not blinded during data collection or analysis. The data collected are not subject to investigator bias.

Reporting for specific materials, systems and methods

We require information from authors about some types of materials, experimental systems and methods used in many studies. Here, indicate whether each material, system or method listed is relevant to your study. If you are not sure if a list item applies to your research, read the appropriate section before selecting a response.

Materials & experimental systems

n/a	Involved in the study
☒	☐ Antibodies
☐	☒ Eukaryotic cell lines
☒	☐ Palaeontology and archaeology
☒	☐ Animals and other organisms
☒	☐ Clinical data
☒	☐ Dual use research of concern
☒	☐ Plants

Methods

n/a	Involved in the study
☒	☐ ChIP-seq
☒	☐ Flow cytometry
☒	☐ MRI-based neuroimaging

Eukaryotic cell lines

Policy information about cell lines and Sex and Gender in Research

Cell line source(s)	Human Embryonic Kidney Cells (HEK293T) were obtained from ATCC.
Authentication	The cell lines used were not authenticated in our laboratories.
Mycoplasma contamination	Cell lines were not tested for mycoplasma contamination.
Commonly misidentified lines (See ICLAC register)	No commonly misidentified cell lines were used in this study.

Plants

Seed stocks	N/A
Novel plant genotypes	N/A
Authentication	N/A
